# Heterozygosity for the Budapest 3 mutation in *SERPINC1* in a family with thrombophilia and structural anomalies of the inferior vena cava

**DOI:** 10.1186/s12959-024-00644-1

**Published:** 2024-08-12

**Authors:** Nina Iversen, Carola Elisabeth Henriksson, Marit Sletten, Marie Skogstad Le, Beate Rikken Lindberg, Rune Andersen, Benedicte Paus

**Affiliations:** 1 Department of Medical Genetics, Oslo University Hospital, Oslo, BOX 4950 Nydalen, N-0424 Norway; 2 Department of Medical Biochemistry, Oslo University Hospital, Oslo, Norway; 3 Department of Cardiothoracic Surgery, Oslo University Hospital, Oslo, Norway; 4 Department of Radiology, Oslo University Hospital, Oslo, Norway; 5 Institute of Clinical Medicine, University of Oslo, Oslo, Norway

**Keywords:** Venous anomalies, Vena cava inferior atresia, Antithrombin deficiency, SERPINC1 Budapest 3 mutation

## Abstract

**Background:**

Atresia of the infrarenal inferior vena cava (IVC) is associated with thrombophilia and antithrombin (AT) deficiency (ATD) due to homozygosity for the so-called Budapest 3 variant, c.391C > T, in the gene, *SERPINC1*.

**Case presentation:**

We report on a father and his two sons that had severe thrombosis at a young age. One son had absence of, and the other had very gracile infrarenal IVC. The father had gracile vena iliaca. All had significant collateral building. AT activity was determined with four different methods and varied between moderately reduced and borderline normal values, depending on the method. While all were heterozygous for c.391C > T, the father was also heterozygous for a variant of uncertain significance in *SERPINC1*.

**Conclusions:**

The findings support the association between c.391C > T in *SERPINC1*, thrombophilia, and atresia of the IVC system and indicate that even heterozygosity for c.391C > T may contribute to such anomalies. ATD detection was hampered by the varying sensitivity of methods used for AT activity measurement.

## Background

Congenital structural anomalies of the venous system are very rare. In anomalies of the inferior vena cava (IVC) two main entities are distinguished [1]. Infrahepatic interruption of the IVC is assumed to be a primary malformation most often seen together with anomalies of other organs like spleen, kidneys, lungs, and heart, reflecting disturbance of complex developmental processes. The primary venous malformation represents an anatomic risk factor for thrombosis. Isolated absence of the infrarenal IVC may be a secondary anomaly that is caused by venous obstruction [2] and except for renal aplasia or hypoplasia other congenital anomalies have not been reported in such patients. Patients with both types of IVC anomalies may be asymptomatic due to development of a venous collateral system, but absence of IVC is associated with high risk for perinatal as well as adulthood deep vein thrombosis (DVT) [3, 4].

In 2015, two cases were reported that indicated hereditary agenesis of IVC [5]. In 2021, De la Morena-Barrio and co-workers were the first to identify a molecular defect involved in agenesis of the IVC system sustaining the role of thrombophilia in agenesis of IVC [6]. They found that severe antithrombin (AT) deficiency (ATD) due to homozygosity for the Budapest 3 variant in *SERPINC1*, c.391C > T was associated with high penetrance of atresia of the IVC system, with absence of the infrarenal IVC. This variant is known to alter the heparin binding site of the *SERPINC1* gene product. The results were considered hypothesis-generating and further research aimed at screening for thrombophilic defects in patients with IVC atresia.

We report on a father and his two sons with a seemingly dominantly inherited mild ATD, the Budapest 3 variant in a heterozygous form, and early DVT. The two brothers had absence and gracility, respectively, of their infrarenal IVC, and the father had gracility of the iliaca vein. All of them had significant collateral building.

## Case presentation

**Fig. 1:**
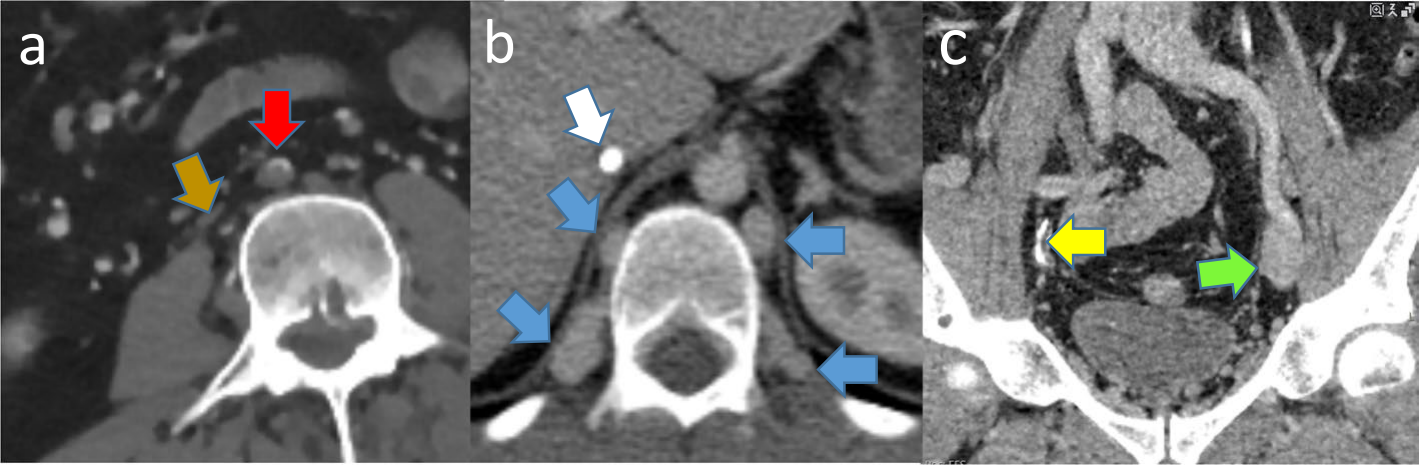
shows contrast enhanced CT scans with coloured arrows indicating important structures. **a** Proband 1 (older brother): Gracile VCI (brown), infrarenal aortic occlusion (red). **b** Proband 2 (younger brother): Calcified reminicents of VCI (white), dilated paravertebral collateral veins (blue). **c** Proband 3 (father): Normal left iliac vein (green). Agenetic/occluded(calcified right iliac vein (yellow)

A boy (Proband 1) with cerebral paresis and spastic paraplegia, epilepsy and significant affection of motoric function suffered DVT in his left proximal femoral vein and right common iliaca vein at the age of 14 years. Computer tomography (CT) venography showed wall changes in the right external iliaca vein, wide, dilated paravertebral veins, and a very gracile infrarenal IVC (Fig. 1a). He was treated with low weight molecular heparin (LWMH) and had surveillance with ultrasound investigations. After a few weeks his anticoagulant treatment was changed to warfarin before switching to apixaban at the age of 18.

The patient’s younger brother (Proband 2) had bilateral DVT after circumcision at the age of 9 years. He had extended thrombosis in both inguinal regions and pelvic veins approaching the intervertebral vessels, in addition to absence of the IVC caudal of the outlet of the hepatic vein and significant collateral development (Fig. 1b). He was first treated with LMWH and subsequently with warfarin. The boys’ father (Proband 3) had a history of lower extremity DVT at the age of 28 years. The parents of Proband 1 and 2 were immigrants from the Republic of Kosovo, and medical records from Proband 3 were not available.

At the age of 22, diabetes mellitus type 2 was diagnosed in Proband 1 and he was admitted to the Department of Dermatology due to purple spots on his feet. Vasculitis was suspected, but at the Department of Vascular Surgery normal venous outlet was found. It was suggested that inactivity due to wheelchair use had contributed to circulatory problems in his lower extremities. Compression stockings and continued treatment with apixaban was recommended. At 23 there was increasing cyanosis of toes with beginning necrosis of the first digit on the left foot. CT angiography of the arteries in the abdomen and pelvis showed gracile aorta and a 63 mm long, total thrombotic occlusion extending from the ostium of the inferior mesenteric artery to the aortic bifurcation. A seemingly atrophic myelolipoma arising from the right adrenal gland was also revealed. Large vessel vasculitis was not found at the positron-emission tomography scan, and no signs of atherosclerosis were indicated. Anticoagulation treatment was changed from apixaban to LMWH. Stenting of the distal aorta was considered too complicated, and a laparotomy with a tube graft was performed. Postoperatively the patient suffered from arterial occlusion in the right inguinal region. Because of critical ischemia the right lower extremity had to be amputated from the femur.

The family was referred to genetic counseling. At this point genome-based analyses were available in our hospital.

## Methods and results

Biochemical thrombophilia screening was performed in all family members in 2023*.* Due to varying levels of AT activity, measurements were carried out with four different commercially available tests, the Innovance Antithrombin and Berichrom Antithrombin III assays (Siemens Healthineers, Marburg, Germany) on a Sysmex CS-5100 analyser (Siemens Healthineers), the HemosIL Liquid Antithrombin assay (Instrumentation Laboratory, Boston, MA, USA) on a ACL TOP 700 analyser (Instrumentation Laboratory), and the STA-Stachrom assay (Diagnostica Stago, Asnières, France) on a STA-R Evolution workstation (Diagnostica Stago) (Table 1). In proband 1, AT activity varied with the different methods from reduced to normal values. In all four assays, AT activity was most reduced in Proband 3, who also had the most reduced antithrombin antigen level. At blood sampling, Proband 1 was treated with LMWH 10 000 IU subcutaneously twice a day, Proband 2 used warfarin, and Proband 3 and the unaffected mother did not take any anticoagulant drugs. In Proband 1, triple-positive antiphospholipid antibodies were present, and they were persistent after 12 weeks.

**Table 1:** Antithrombin analysis

		Antithrombin activity (IU/dL)			Antithrombin antigen level (%)
Method	Innovance HemosIL Quantikine	STA-Stachrom	Berichrom	CI/antithrombin-III	
	Xa based	Xa based	Ila based	IIa based	ELISA R&D
Normal range	80–120	83–128	80–120	80–120	70–131
Proband 1	63	78	79	81	75
Proband 2	51	76	74	77	67
Proband 3 (affected father)	45	52	56	60	51
Unaffected mother	102	112	114	98	79

Proband 1 and 2 had previously been radiologically investigated. CT venography in proband 3 was performed and showed normal IVC. Other findings, including gracility of the vena iliaca and a significant collateral system, were interpreted as post-thrombotic changes (Fig. 1c).

All family members tested negative for the common factor V Leiden and the prothrombin G20210A mutation in the *F5* and *F2* genes. Whole exome sequencing (WES) was carried out in Proband 1 and 2 to identify possibly contributing factors to their IVC anomalies and a genetic cause for the observed reduced AT activity. WES revealed heterozygosity for the Budapest 3 mutation, c.391C > T, p.Leu131Phe in the *SERPINC1* gene in both brothers. Several loss-of-function and missense variants with a frequency below 0.1% were observed in genes with hitherto unknown connections to the phenotype. In Proband 1, an exome-based gene panel for thrombophilia comprising 135 genes, was also analyzed in an accredited diagnostic laboratory, confirming the previous finding of the Budapest 3 mutation but with no further finding of pathogenic variants. The exome-based gene panel for vascular malformations was performed for Proband 3, with negative results.

We verified the WES results by Sanger sequencing of the entire *SERPINC1* gene and included all family members. Heterozygosity for the AT Budapest 3 variant was confirmed in all probands but not in the asymptomatic mother. Of note, Proband 3 was also heterozygous for the variant, c.1063T > G (p.Phe355Val) in *SERPINC1*. The entire *F2* and *F11* genes were also Sanger sequenced and no relevant findings were detected in the three probands.

## Discussion and conclusions

Inherited antithrombin deficiency (OMIM#613,118) [7] was indicated in this family, and heterozygosity for the Budapest 3 mutation in *SERPINC1* could explain the reduced AT activity in the three probands. To detect the Budapest 3 mutation in the heterozygous form the Innovance was the most sensitive, and the Berichrom the least sensitive assay, which is in line with the findings of Rojnik et al [8]. The Berichrom assay could not detect the Budapest 3 variant in its heterozygous form in Proband 1, and the HemosIL and STA-Stachrom assays resulted in borderline values. Thus, in some cases, the use of these three reagents may not detect the heterozygous Budapest 3 variant and lead to underdiagnosed ATD [8]. It could be speculated that the reduced AT activity and antigen levels in Proband 3 was caused by the combination of the Budapest 3 mutation and the variant, c.1063T > G, in *SERPINC1*, as the ATD patient reported by Provaznìkovà and co-workers [9] carried the same combination of variants. However, although predicted to alter a well-conserved amino acid, the latter variant was interpreted as of uncertain clinical significance according to the ACMG/AMP criteria [10]. Furthermore, the sons of Proband 3 had earlier and more severe clinical presentation than their father and did not carry the variant, C.1063T > G.

The finding of Budapest 3 mutations in this family is in line with the association between this variant, thrombophilia, and isolated infrarenal IVC anomalies, although the previously reported cases were homozygous for this variant [6]. Indeed, heterozygote patients were not included in the study of De la Morena-Barrio and co-workers. Their finding of reduced penetrance of structural IVC anomalies even in homozygote individuals is in line with this report.

Heterozygosity for the Budapest 3 mutation may partly explain the observed severe thrombophilia and IVC anomalies. In proband 1 antiphospholipid syndrome and paraplegia with underdeveloped and hypoplastic arteries may also have contributed to arterial insufficiency as well as arterial and venous thrombosis. The cause of his cerebral paresis is not known, although discolored amniotic fluid and possible neonatal asphyxia were mentioned in his medical records. We did not find that a cerebral MRI was performed. A perinatal vascular event cannot be excluded. The severity of the collective clinical history in this family in the presence of only modest reduction in AT activity urged us to carry out further searches for other contributing genetic causes. However, despite comprehensive genomic investigations, no indication of digenic or autosomal recessive inheritance of the sons’ IVC anomaly with a hitherto identified maternal genetic contribution was found. Contribution from yet unidentified genetic factors cannot be excluded.

In this family pre- or postnatal venous obstruction is likely to have caused structural anomalies of the IVC. Our results may indicate that even heterozygosity for the Budapest 3 mutation and moderately decreased AT activity may have contributed to atresia of the inferior IVC system. In severe familial DVT or IVC anomaly, choice of method for determination of AT activity or genetic testing of the *SERPINC1* should be considered if laboratory thrombophilia screening test results are within reference ranges. Identifying thrombophilic risk factors in structural IVC anomalies is important and may contribute to prevention of severe complications later in life.

## Data Availability

As all investigations were considered as part of clinical diagnosis, all results were stored as the patients’ individual medical records which are subject to general data regulation and protection. Selected anonymous data can be available upon request to the corresponding author.

## Abbreviations

ACMG: American College of Medical Genetics and Genomics
AMP: Association of Molecular Pathology
AT: Antithrombin
ATD: Antithrombin deficiency
CT: Computer tomography
DVT: Deep venous thrombosis
IVC: Inferior vena cava
LMWH: Low molecular weight heparin
WES: Whole exome sequencing

## Data availability

No available datasets were generated or analysed during the current study.
